# Interfacial reaction during friction stir assisted scribe welding of immiscible Fe and Mg alloy system

**DOI:** 10.1038/s41598-021-81266-9

**Published:** 2021-01-15

**Authors:** Hrishikesh Das, Piyush Upadhyay, Tianhao Wang, Bharat Gwalani, Xiaolong Ma

**Affiliations:** 1 Applied Materials and Manufacturing Group, Pacific Northwest National Laboratory, Richland, WA USA; 2 Physical and Computational Sciences Division, Pacific Northwest National Laboratory, Richland, WA USA

**Keywords:** Metals and alloys, Transmission electron microscopy

## Abstract

We report on interfacial characteristics and chemistry of bonded Mg-Fe interfaces welded using friction stir assisted scribe technique (FaST). Two pairs of dissimilar joints: (AZ31-DP590) and (Pure Mg-DP590) were studied to shed light on joining mechanisms responsible for bonding of “immiscible” pairs of Mg and Fe. We present first direct experimental evidence of presence of oxide layer, Al segregation by atom probe tomography and nano steel grains close to interface by transmission electron microscopy study.

## Introduction

Mg and Fe are considered immiscible because they have extremely low solubility in each other, which makes joining them challenging^1^. Efforts have been made using bridging interlayers such as Ni^2^, Cu^3^, Sn^4^, or Zn coating on the steel^5^ or a Mg-based filler^6^ to intentionally introduce metallurgical bonding at the interface during welding. A few emerging methods that employ high shear and shear rate at the interface have demonstrated viable bonds between Mg and Fe systems with and without the need for alloying elements. Attempts have been made to understand the underlying mechanisms responsible for bonding based on these interfacial observations. A better understanding of bonding mechanisms will help engineers improve desirable joint properties. Typically, in the presence of Al, the bonding has been attributed to the presence of various Al–Fe intermetallic compounds (IMCs) found at the joint interface. IMCs of thicknesses ranging from 100 nm to 10 µm have been found at the interface that may facilitate lattice matching between Mg and Fe systems. Kasai et al. (friction stir welding (FSW) butt joint)^7^ and Xu et al. (resistance spot welding (RSW) joint)^8^ attributed bonding of Mg/steel to formation of 1.5 µm or thinner Fe_2_Al_5_ at the interface. Liu et al.^9^ found nanoscale epitaxial growth of an Al–Fe (Al_3_Fe) intermetallic layer in resistance spot-welded AZ31-DP 600 joints. When Al was absent, no distinct compound was observed at the interface. The underlying mechanism in this case is not well studied. A Mg nano-grain layer (100–200 nm) at the interface was attributed as a coupling layer between bulk Mg and bulk Fe by grain boundary relaxation^9^. Yet, bonding has also been reported in welding processes (friction stir assisted scribe technique [FaST], ultrasonic, impact welding) that do not exhibit any sign of bulk melting^10,11^.

In this work, we reveal interfacial chemistry of a Mg/steel interface produced by FaST using multimodal characterization approach to broaden our understanding of welding mechanisms in immiscible systems. Details of FaST welding are provided in Supplementary Fig. S1. We used two specific combinations: (a) AZ31 Mg with uncoated DP 590 steel and (b) pure Mg with uncoated DP 590 steel. The Zn layer was chemically removed from the as-received DP 590 to avoid Mg/Zn interaction and eutectic formation at the interface^12^. In case (b), commercially pure elemental Mg^13^ was used to avoid the effects of alloying elements. Transmission electron microscopy (TEM) and energy-dispersive x-ray spectroscopy (EDS) analysis together with atom probe tomography (APT) were conducted at the interface.

## Results and discussion

**Figure 1:**
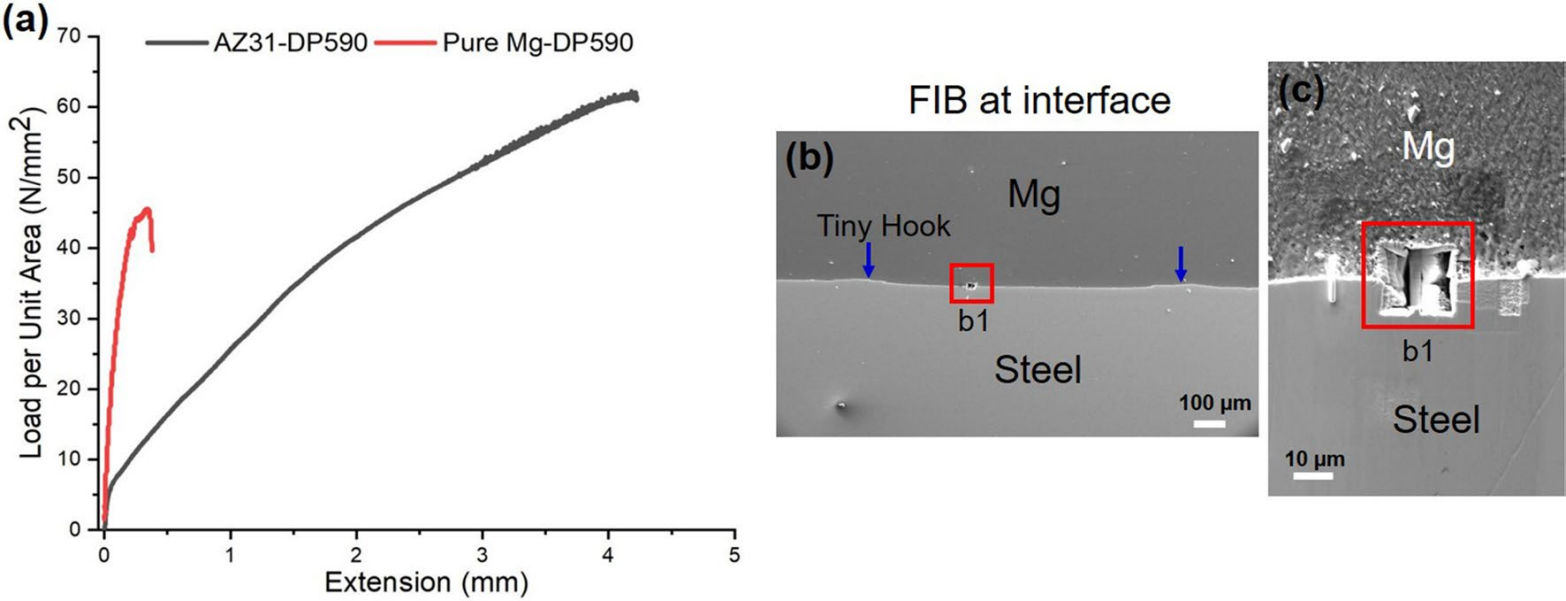
Joint strength and FIB. (**a**) Load per unit area VS extension plot for AZ31-DP 590 and pure Mg-DP 590 joints; (**b**) and (**c**) FIB micrographs at interface.

Figure 1(a) shows representative lap shear strength of the two weld cases investigated in this study. Both the joints fractured at the interface; thus, the weld strength is a direct measure of the weld’s load bearing capacity expressed in terms of force per unit joint area. The data in Fig. 1(a) indicates that the joints are well bonded. The AZ31-DP 590 joint shows strength close to 50% of that of AZ31 base metal and the pure Mg-DP 590 joint also shows reasonable joint strength despite the poor strength of pure Mg base metal. A typical scanning electron microscopy (SEM) cross section at the interface (Fig. 1(b)) shows the interface bonded area (inside solid rectangular outline). In comparison to typical FaST joints^12^, smaller plunging depth resulting in ~ 40 μm of scribe engagement was used during welding to ensure interfacial fracture. Thus, the characteristic steel hook feature is significantly reduced. TEM foils were extracted from the vicinity of the interface region (Fig. 1 b,c) for both AZ31-DP 590 and pure Mg-DP 590 welds, using a focused ion beam (FIB) lift-out technique (details are provided in the Experimental Methods). Cautions were taken to thin the heterojunction interface to make both sides electron transparent while retaining the interface integrity.

### TEM analysis at the interfaces of AZ31-DP 590 joint and pure Mg-DP 590 joint

**Figure 2:**
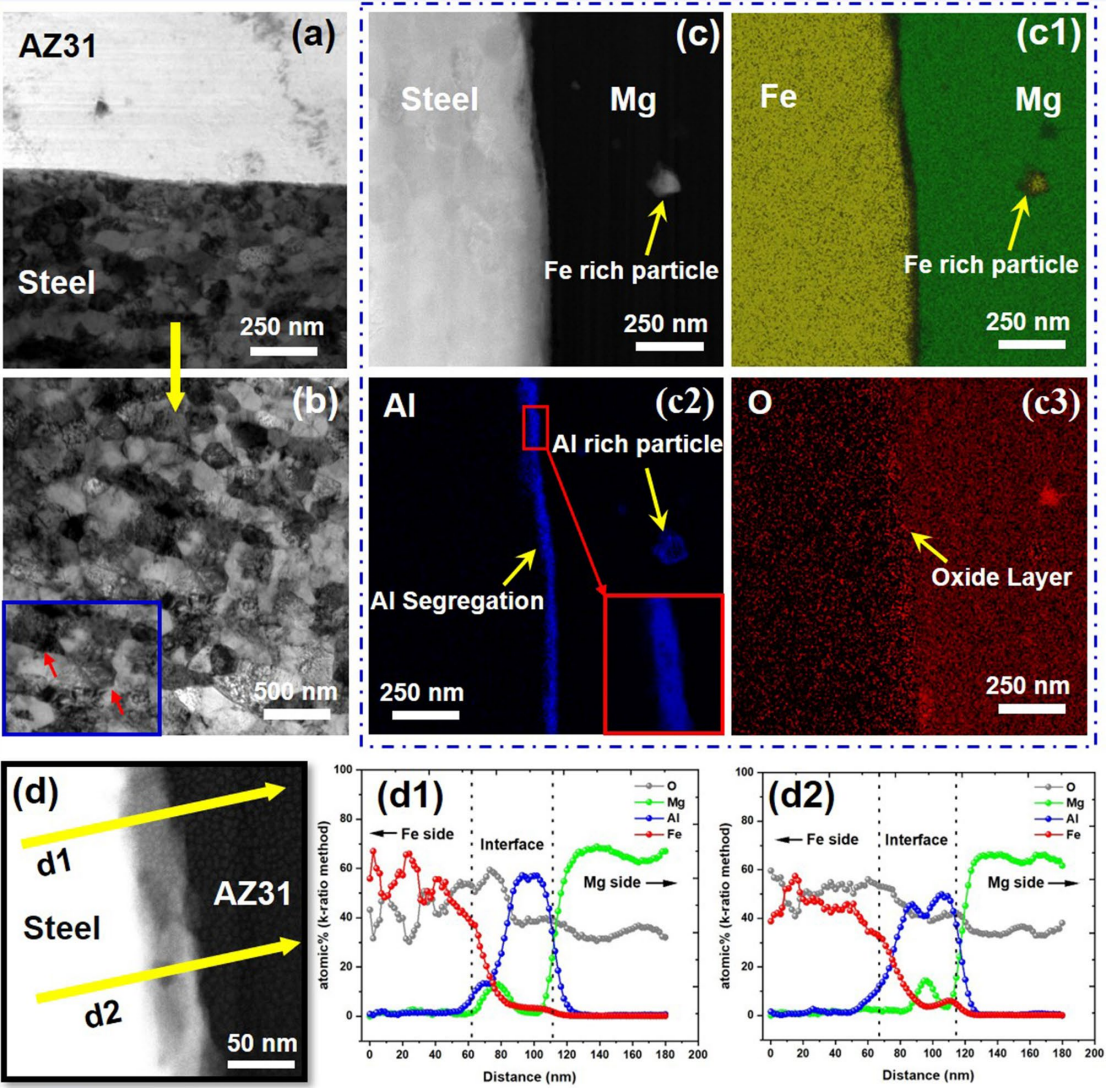
TEM with EDS of AZ31Mg-DP 590 joint. (**a**) Joint interface; (**b**) nano steel grains (inset: dislocations marked by red arrows); (**c**) S/TEM image, EDS mapping of (c1) Fe and Mg, (c2) Al (inset: high magnification view), (c3) O; (**d**) location of two EDS line scans; EDS line scan elemental analysis plots (d1) and (d2), for AZ31Mg-DP 590.

**Figure 3:**
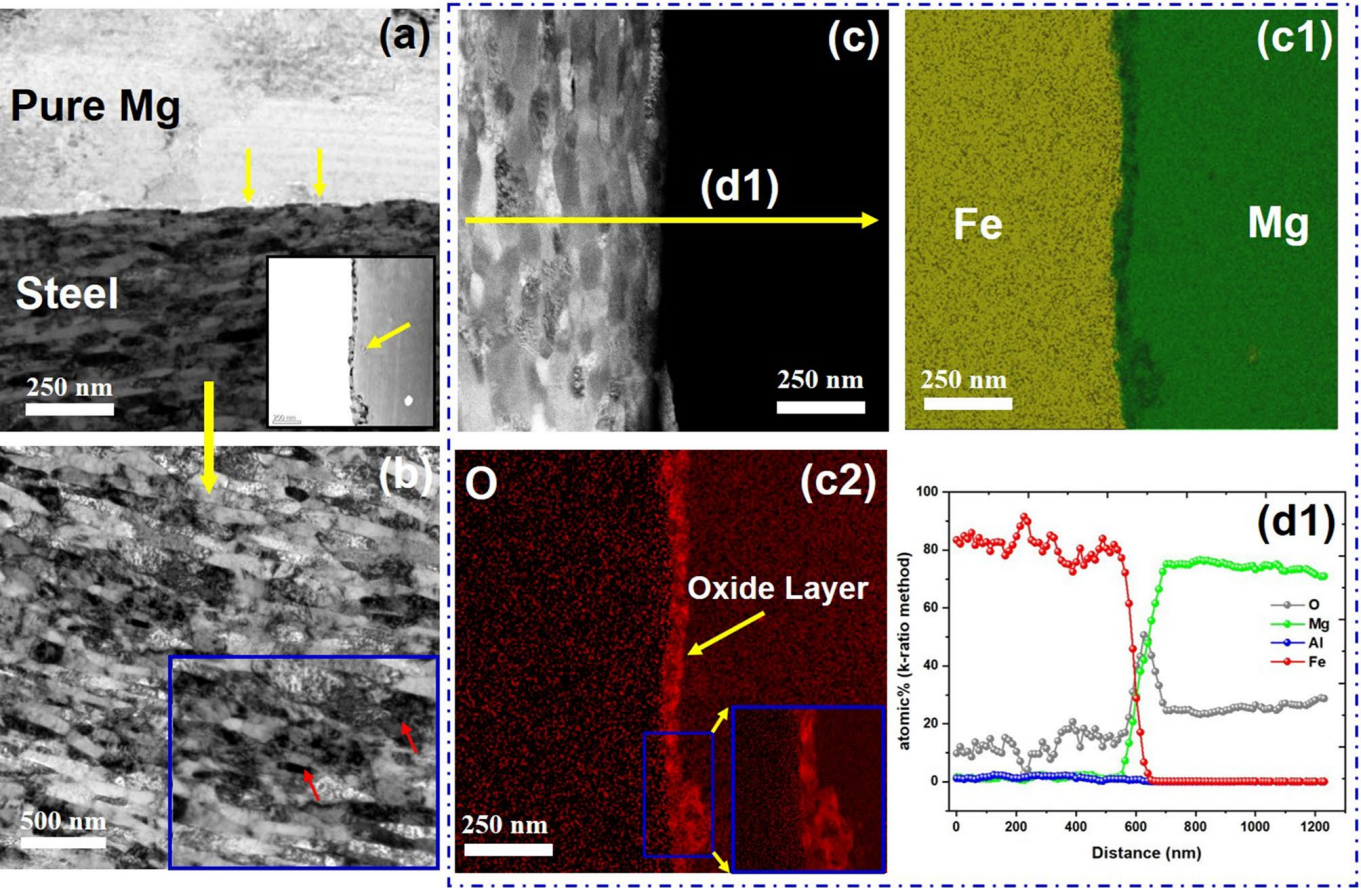
TEM with EDS of pure Mg-DP 590 joint. (**a**) Joint interface (inset: high-contrast, high-angle annular dark field image with oxide layer at the interface); (**b**) nanosized steel grains (inset: dislocations, marked by red arrows), (**c**) S/TEM image with location of EDS line scan; EDS mapping of (c1) Fe and Mg, (c2) O (inset: high magnification view), (d1) line scan elemental analysis plot for pure Mg-DP 590.

The TEM bright-field micrographs of joint interfaces and the steel side for AZ31-DP 590 steel and pure Mg-DP 590 steel welds are presented in Figs. 2(a, b) and 3(a, b), respectively.

Owing to severe plastic deformation (SPD) from the scribe cutting on the steel surface, ultrafine grains (UFGs) formed on the steel side next to the welded interface. Grains of steel were significantly refined from 8 ± 5 µm (base steel) to 200 ± 100 nm in the AZ31-DP 590 joint and to 70 ± 15 nm for the pure Mg-DP 590 steel joint. The grain size appears to gradually increase with distance from the interface. Nevertheless, the refined grains span 37 ± 3 µm away from the interface (Supplementary Fig. S2), indicating the extent of the plastic deformation in steel beyond the observed scribe engagement.

Mg side, where conventional FSW occurred also contained recrystallized and refined grains. The extent of grain refinement was much less than on the steel side. The grain size reduced from 20 ± 10 µm to 3 ± 2 µm. This could be associated with the comparatively high processing temperature in Mg, resulting in greater grain growth after recrystallization than in steel. Scanning transmission electron microscopy (S/TEM) and EDS elemental maps at the interface and surrounding area are shown in Fig. 2(c–c3). An iron-rich particle of approximately 100 nm size was observed ~400 nm away from the interface on the Mg side. This is likely a result of the scribing, similar to what has been routinely observed in macro cross sections in previous FaST work^12^. Nevertheless, this is a first observation of nano sized stray steel particles in FaST welding.

At the interface, a continuous, nanosized, Al-rich layer (~ 40 nm thick) was found (see the high magnification inset in Fig. 2(c2)). This suggests formation of an IMC, which has been previously reported in butt welding of Mg/steel albiet at much higher micron level thickness^7^. Al-rich chemistry around the stray steel particle on the Mg side shows that conditions are favorable for the formation of Al/Fe IMC layer. Fe- and Al-rich peaks also matched well (line scan d2) indicating formation of an Al–Fe IMC at the interface^7^. An appreciable amount of O was also observed, especially towards the Mg side and the interface region. From line scan d1 and d2 (Fig. 2d), the elemental gradient of Mg and O suggests the presence of MgO layer at the interface.

S/TEM with EDS elemental mapping of pure Mg and DP 590 steel is presented in Fig. 3(c–c2). In the absence of Al, the interface layer has a distinct composition. An O-rich interfacial layer with profuse nanosized lumps (Fig. 3c2) was identified close to the interface on the Mg side. A high-contrast view at the interface further reveals the nanosized lumps (seen as darker spots, marked with yellow arrows in the Fig. 3(a) inset). This layer is ~ 35 ± 5 nm thick and is present across the interface (see the high magnification inset in Fig. 3(c2)). An EDS line scan also suggests the formation of a nanosized MgO layer (Fig. 3(d1)).

A good grasp of interfacial phenomena is critical for understanding the joint chemistry and controlling them for optimum joint performance. The following paragraphs describe our attempts to understand the interface chemistry based on the presented results.

Materials combination and lattice matching determine coherence or lack thereof between two phases or across an interface^14^. For any metallurgical bonding, local temperature is also an important factor. Measured peak temperature at the AZ31-DP 590 interface was around 550 °C (Supplementary Fig. S2(e)), which is enough to enhance the up-hill Al diffusion towards the interface (Al melting point 660 °C)^15,16^. While it is tempting to draw from the existing knowledge base for elemental diffusion across an interface at high temperature and pressure, the time scale of the welding process is a few orders of magnitude lower than in a typical diffusion experiment. For example, in FaST a 1 mm interface is directly exposed to the heat source for 0.08 s. Also, the total sheet thickness is 3 mm, so a much higher cooling rate would be expected. Therefore, the interfacial chemistry is more localized and could vary slightly with location as well^17,18^. It is likely that Al diffused from the Mg side towards the interface in response to high temperature, pressure and to a negative heat of mixing with Fe. Owing to high welding speed, short diffusion time, and weak diffusion coupling of Al towards Fe, a nanosized Al rich layer (40 ± 10 nm) formed at the interface^12,18,19^. Al has excellent coherence with both Mg and steel facilitating its intrusion into  the interface as a coupling layer. It is well known in the field of dissimilar joining that thin microscale to nanoscale interface layers enhance the joint strength^19,20^.

It is well known that elemental magnesium is very unstable and prone to surface oxidation even at room temperature. Regardless of how clean the surface is, a small layer of oxide is bound to form in when exposed in air. Thus, it is valid to expect that just prior to joining the Mg surface contained a thin layer of MgO (~ 30 nm^21^). This “prior” oxide layer however will not go undisturbed during the welding process as a significant amount of severe plastic deformation occurs at the interface due to the scribe cutting action. Additionally, a lot of material transport takes place around the interface. EDS elemental distribution (Fig. 2 (d1) and (d2), Fig. 3 (d1)) clearly indicates a significant saturation of oxygen at the interface compared to nearby processed zone. This cannot be explained by prior surface oxides/ hydroxides in the Mg side alone. We posit that oxygen saturation at the interface is a direct result of the joining process. Specifically, for pure Mg- DP590 case, in absence of Al (unlike the AZ31-DP590 case), we hypothesize that joining of Fe to Pure Mg may be mediated by lattice matching of a very thin atomic layer of oxides. Given that no possible lattice plane matching exists for Mg/Fe coupling according to the edge-to-edge matching model for the hexagonal closest packing/body centered cubic system^22^. However, a large extent of lattice matching (with only a small lattice mismatch of 3.8%) on a 45° in-plane rotation can be found between MgO and Fe, arising from strain misfit by dislocations^23,24^. FaST induced Severe plastic deformation could introduce lattice mismatch between Fe and MgO to some extent. Thus, the nanosized oxide layer may act as a coupling between two immiscible systems. Previous research work using impact welding have also shown higher oxygen concentration at the interface (Al-Steel joints) and also shows oxygen over saturation can take place at the interface^25^.

For both AZ31-DP 590 and pure Mg-DP 590 joints, nanosized lath martensite formed (200 ± 100 nm and 70 ± 15 nm, respectively) in a texture pattern close to the interface with accumulation of dislocation, as shown in Figs. 2(b) and 3(b), respectively (dislocations are marked with red arrows). SPD induced by the scribe along with a high cooling rate transformed an island of martensite (in the DP 590 base metal microstructure) into nanosized laths (UFGs) with a highly strained grain boundary. This effect enhances the rapid dislocation pileup and interactions at the grain boundaries^26–28^, as shown in Figs. 2(b) and 3(b). Because the relief of misfit strain by high energy nano laths enhances the grain boundary relaxation, activation energy for reaction is reduced^9,29,30^. Previously researchers also observed nanolayer formation (Mg–Mn)^31^, and grain boundary relaxation phenomenon (Mg–Fe)^9^ acting as a coupling between two immiscible systems.

### Atom probe tomography

**Figure 4:**
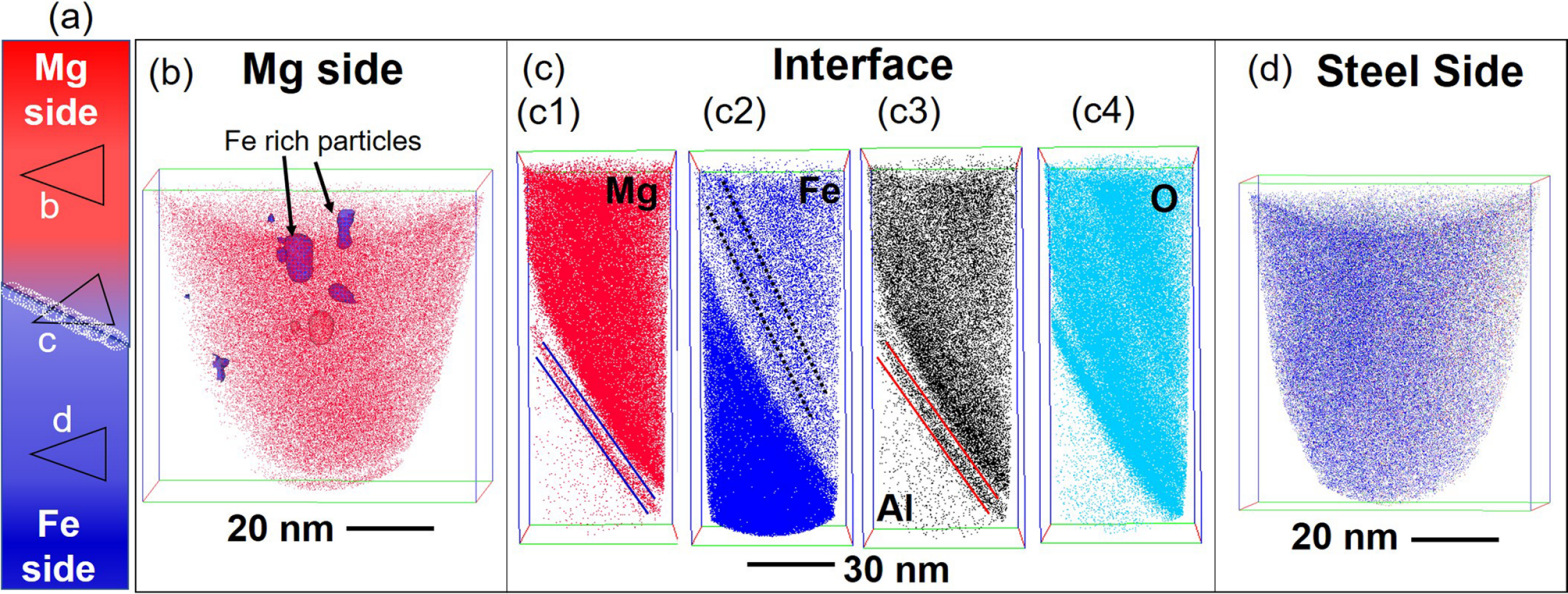
APT results from the interfacial region of the FaST joint between AZ31 and DP590. (**a**) schematic showing the region of interest for the APT analysis (notation **b**, **c** and **d** indicates corresponding figures) ; (**b**) reconstruction showing the Mg ion map from Mg alloy side; (**c**) reconstructions showing the Mg, Fe, Al, and O maps including the interface between the two alloys; (**d**) DP 590 steel side.

**Figure 5:**
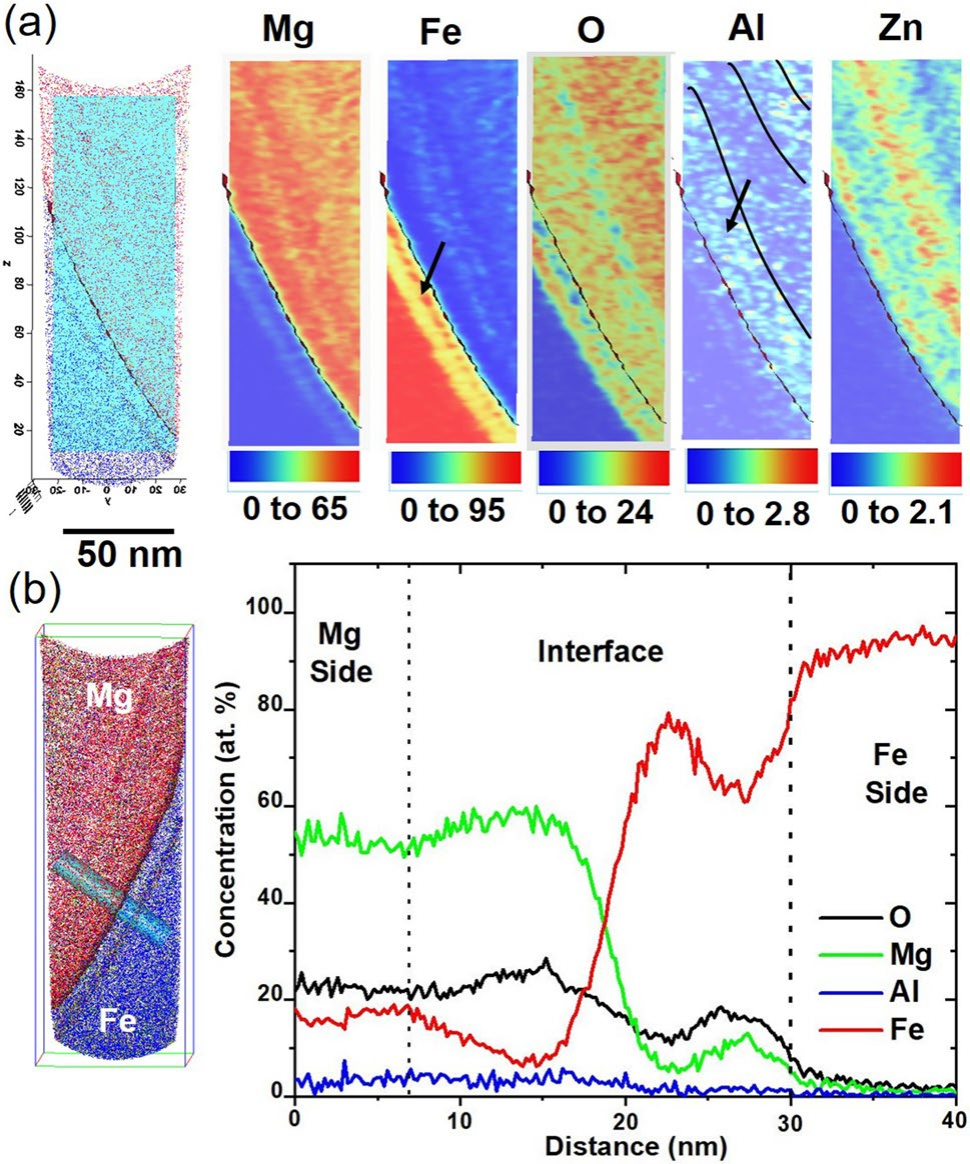
Elemental distribution across the interfacial region of the FaST joint between AZ31 and DP590. (**a**) Two-dimensional compositional map of the Fe, Mg, O, Al and Zn; (**b**) one-dimensional composition change along a cylinder 10 nm in diameter and 60 nm long.

For additional insight into the elemental diffusion mechanism, APT was performed at the interface of AZ31-DP 590 steel (Fig. 4). The needle shaped specimens for APT analysis were made from the welded region such that a few needles captured the composition of the welded region on Mg side, interface, and Fe side, as shown in Fig. 4(a). Figure 4(b) shows the reconstruction depicting the Mg ions (red) from the needle made from the AZ31 side (~ 1 µm away from the interface). A few discrete Fe-rich regions are evident in the reconstruction, as highlighted by the arrows in the figure. These regions were mechanically transferred to the Mg side by the scribe action during the FaST process. While micron sized stray steel particles in the Mg side has been reported previously^12^, this is the first observation of nano-sized steel particles on the Mg side in FaST process. Additionally, the overall region on the Mg alloy side is enriched to ~ 10 at.% Fe. The APT results from the interfacial region (Fig. 4 (c1–c3)) confirmed the formation of a nanoscale Al-rich layer along with oxygen enrichment. Note that the reconstructions of Mg, Fe, Al, and O in Fig. 4(c) show nanolayers of compositionally distinct regions, indicating likely mass transfer during welding. For example, note a layer rich in Mg, O, and Al, highlighted by parallel blue lines in the Mg map, a Fe-rich layer and Al rich layer are highlighted by black dotted lines and red lines in respective Fe map and Al map. The quantitative assessment of the elemental gradient across the interface is presented in Fig. 5. The reconstruction from the DP 590 steel side with the Fe ions in blue (Fig. 4(d)) shows a homogeneous distribution of elements with a minimal Mg enrichment resulting from welding.

APT of a 5 nm slice of the needle excised from the interfacial region is given in Fig. 5 (a). A rectangular area of 150 nm × 50 nm × 1 nm was selected to construct two-dimensional compositional maps of the Fe, Mg, O, Al and Zn to examine their local distribution across the interface. APT compositional map at the interface shows no trace of leftover Zn on the Steel side.

Interestingly, the O concentration is high (up to 24 at.%) throughout the alloy on the Mg alloy side. The Al-rich layer is delineated in the Al maps, where the local Al content reaches ~ 2.4 at.%, which is much lower than was observed in our TEM analysis (Fig. 2). In the TEM analysis, we noticed that Al concentration can be as high as ~ 40 at% in several locations. However, based on a combined assessment of TEM and APT results, it can be concluded that a complex oxide forms near the interface, with regions enriched in Al and Mg. It is likely that the FIB sample used for APT analysis contained Mg-enriched areas, while in TEM (at a relatively larger scale) we observed the overall distribution of the Al–Mg and O elements.

The oxygen concentration across the interface and on the Mg side is limited to ~ 25 at%, which, however, can also be an underestimation. A one-dimensional composition change along a cylinder 10 nm in diameter and 60 nm long is plotted in Fig. 5(b). The local modulations in the composition are clearly captured in this plot because the base metal and the weld are nanolayered. Enrichment of Fe concentration towards the Mg side supports the concept of mechanical alloying or mixing induced by SPD^32^. Hence, it is very likely that high strain and shear during SPD enhances the abundant vacancies (point defects) and dislocations (line defects) so that a complex MgO, (Mg–Al) O type oxide is formed at the interface^33^. However, the lattice mismatch renders it difficult to form a homogeneous nanolayer throughout the interface. Rather, it is a more localized phenomenon and depends on the local atom and lattice misorientation^25^. The structure of the oxide and structural changes at the interface will be confirmed using high resolution TEM analysis being currently pursued by the authors, which is outside the scope of the current paper.

## Concluding remarks

We studied the interfacial chemistry of two pairs of Mg-Fe dissimilar joints made using Friction stir assisted scribe technique. In the case of AZ31-DP590, the interface was mediated by a nano scale thin layer of segregated Al. In addition, a complex Al/Mg oxide layer is also found at the interface. In the case of pure Mg-DP590, the interface consists of nano scale oxygen rich layer which may act as a bridging layer between the two immiscible system via lattice matching. The Mg and Fe region immediately next to the interface consists of ultra-fine/ nano grains which may also be contributing to bonding via misfit strain relief through grain boundary relaxation.

## Experimental methods

Sheets of AZ31 Mg (2 mm thick), pure Mg (1 mm thick), and DP 590 steel (1 mm thick) were used for these lap joint assemblies. All the weldings were performed in position control mode. To measure the temperatures at the weld interface, a thermocouple was inserted through the tool body such that it was flush at the pin tip (detailed welding process parameters and tool feature are presented in Supplementary Table S1, Supplementary Table S2, and Supplementary Fig. S1b). The microstructures at the weld interface were characterized using SEM, TEM, S/TEM, and APT. The SEM imaging and the sample preparation for S/TEM and APT (using the FIB lift-out technique) were performed using a Thermo Fisher Scientific Quanta 200 FIB-SEM outfitted with an Oxford Instruments EDS system for compositional analysis (detailed APT techniques are presented in Supplementary Fig. S3). A FEI Titan 80-300 operated at 300 kV and a JEOL ARM200F operated at 200 kV were used for S/TEM. A CAMECA LEAP 4000X HR APT was used in pulsed voltage mode at 200 kHz pulse frequency, with 20% pulse fraction, and a specimen temperature of 50–60 K, while the detection rate was maintained at 0.005 atoms/pulse. In accord with Standard ASTM D1002-10^34^, transverse lap shear tensile samples 30 mm wide × 150 mm long were cut by electrical discharge machining^12^ from both AZ31-DP590 and pure Mg-DP 590. Unguided lap shear tensile tests were conducted with a cross head speed of 76.2mm/s using an Instron 8860 universal tensile testing machine.

## Supplementary Information

Supplementary Information.
