# Supplementary material for: Interfacial reaction during friction stir assisted scribe welding of immiscible Fe and Mg alloy system
Source: Sci Rep. 2021 Jan 15;11:1588. doi: 10.1038/s41598-021-81266-9 (PMC7810708; doi:10.1038/s41598-021-81266-9)
Supplement: Supplementary file 1 — Supplementary Information. [file 41598_2021_81266_MOESM1_ESM.docx]

**Supplementary Information**

**Interfacial reaction during friction stir assisted scribe joining of immiscible Fe and Mg alloy**

Hrishikesh Das^a^, Piyush Upadhyay^a*^_,_ Tianhao Wang^a^, Bharat Gwalani^b^, Xiaolong Ma^a^

*^a^Applied Materials & Manufacturing Group, Pacific Northwest National Laboratory, USA*

*^b^Physical and Computational Sciences Division, Pacific Northwest National Laboratory, USA*

^*^Corresponding author: [Piyush.Upadhyay@pnnl.gov](mailto:Piyush.Upadhyay@pnnl.gov)

**1. “FAST” process**

In the general concept of lap joining dissimilar materials, the low melting point material (here Mg) is always placed on top (Supplementary Fig. S1a). Conventionally, the FSW pin plastically deforms the top sheet (lower melting point material) [1-3]. In a similar fashion, a scribe is attached axis symmetrically to the tip of the pin to cut or deform the bottom sheet (high melting point material, here steel) (Supplementary Fig. S1b). As the tool rotates, a mechanically interlocking (hook) feature is generated at the interface. The depth of cut in the bottom layer is determined by the length of the scribe cutter. A predetermined gap between the tip of the FSW pin and the interface is maintained so that only the scribe cutter is in contact with the bottom steel sheet while the FSW tool interacts with the top Mg sheet [3]. The FAST tool is made from heat-treated H13 tool steel, while the inserted scribe is made from a cobalt drill bit blank (Supplementary Fig. S1b).

A representative tool feature with scribe and thermocouple insertion locations and FAST welded AZ31-DP 590 and Pure Mg-DP 590 lap joints are shown in Supplementary Fig. S1 (b), (c), and (d), respectively. Detailed FAST tool geometry and the optimized welding process parameters are listed in Supplementary Table S1 and Supplementary Table S2, respectively. The slightly lower rotation rate used for pure Mg-DP 590 joints accommodates the softening nature of the pure Mg: a high rotation rate induced higher heat input, resulting in defects at the surface and the interface. Temperature profiles for two different joints are exhibited in Supplementary Fig. S1(e). The welding length for AZ31-DP 590 and pure Mg-DP 590 joints were 275 mm and 80 mm, respectively. The galvanized steel was dipped in 5% HCl acid solution for 30 minutes dissolving any trace of Zn coating from the steel surface.

Supplementary Table S1. Dimensions of the FAST and FSW tools applied in this study (mm)

| Material combination | Shoulder diameter | Pin diameter | Pin length | Scribe diameter | Scribe length | Scribe offset |
| --- | --- | --- | --- | --- | --- | --- |
| AZ31-DP590 | 12 | 5 | 2.3 | 0.3 | 0.35 | 1.5 |
| Pure Mg-DP 590 | 12 | 5 | 1 | 0.25 | 0.35 | 1.5 |

Supplementary Table S2. Welding parameters for FAST

| Material combination | Welding parameters | | | |
| --- | --- | --- | --- | --- |
|  | Rotation rate (rpm) | Traverse speed (m/min) | Tilt angle (degrees) | Plunge depth (mm) |
| AZ31-DP590 | 1,950 | 0.75 | 1 | 2.05 |
| Pure Mg-DP 590 | 1,000 | 0.5 | 1 | 1.15 |


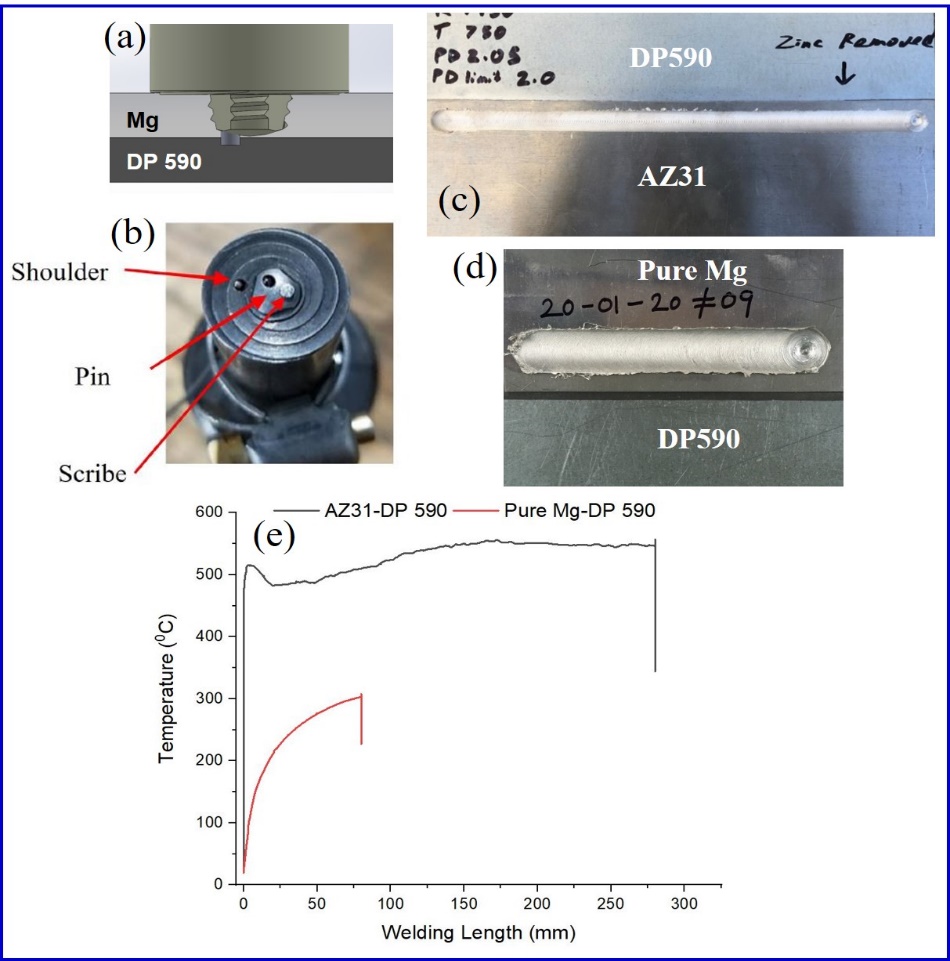


Supplementary Fig. S1 (a) Schematic of FAST process, (b) top view of FAST tool, (c) AZ31-DP 590, and (d) Pure Mg-DP 590 lap joint made by FAST, (e) Measured temperature comparison for AZ31-DP 590 and pure Mg-DP 590 joints.

**2. SEM analysis at the interface**

The refined grains span 37 ± 3 µm away from the interface (Supplementary Fig. S2), indicating the extent of the plastic deformation beyond the scribe engagement.


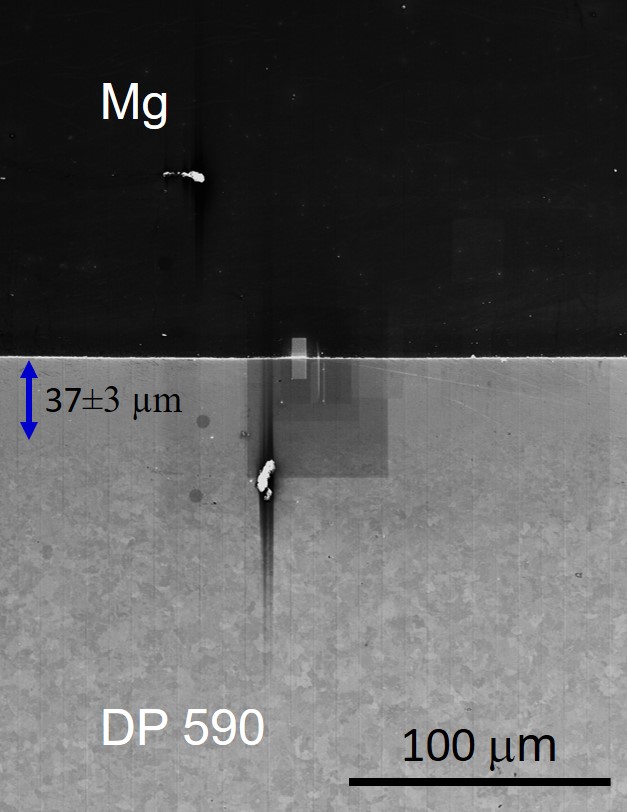


Supplementary Fig. S2. Nanosized refined grains span 37 ± 3 µm away from the interface.

**3. Atom probe tomography of the interface between AZ31 and Steel**

For the successful field evaporation of the needles in APT, the FIB lifted samples were rotated, 90 degrees out of plane and then 45 degree in plane in the FIB before making the needle specimen. As the field evaporation energies of Fe (33 V/nm) and Mg (21 V/nm) are very different, the plane by plane field ionization of both Mg rich side and Fe rich side was not seemingly possible with the interface in the vertical direction. Hence, the we rotated the lifted out micro slab using the omni probe manipulator in the FIB and aligned the interface at 45 degree to the field ionization axis. This allowed a smooth transition from Fe rich side to the Mg rich side.


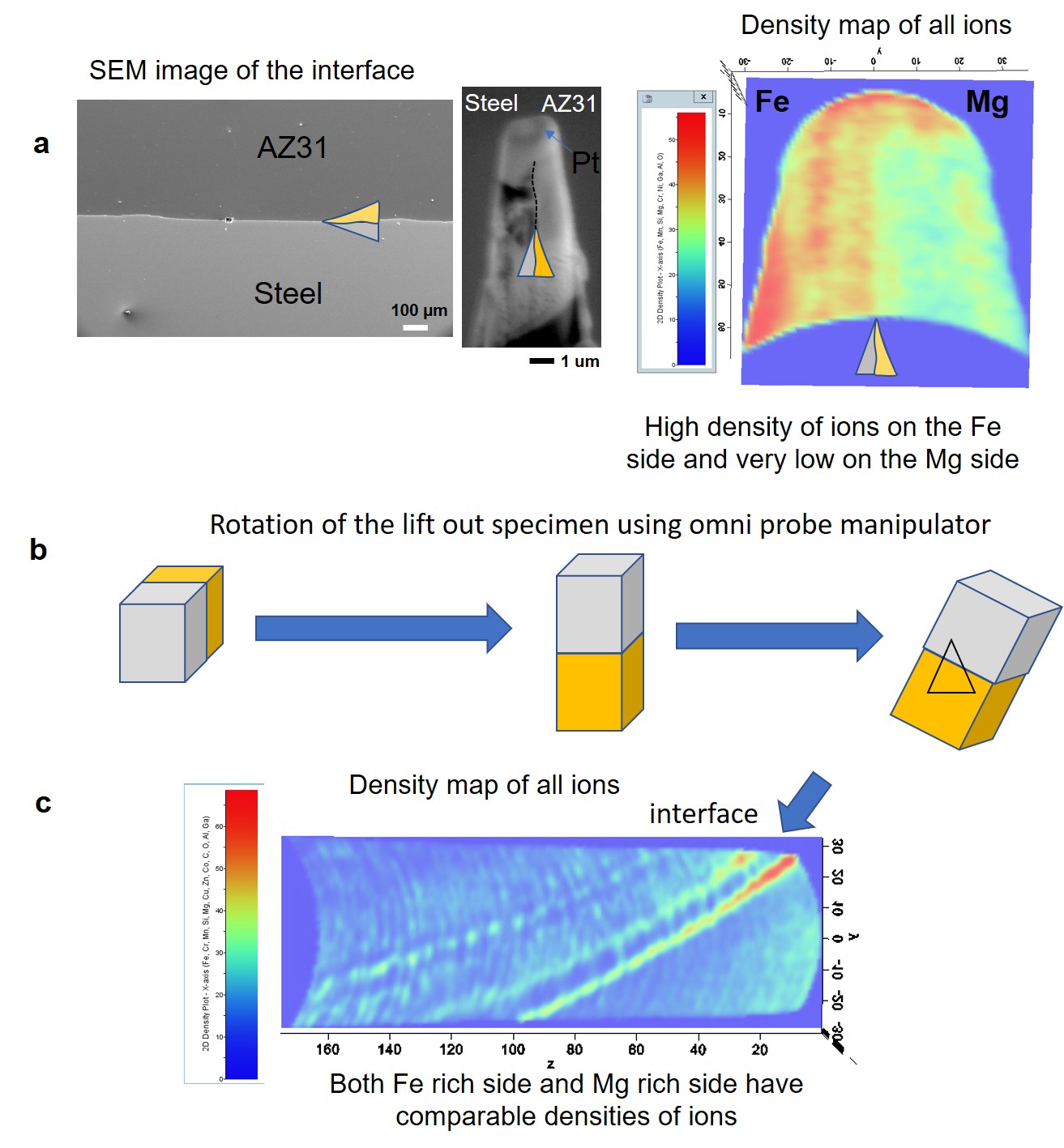


Supplementary Fig. S3. Atom probe tomography of the interface between AZ31 and Steel. (a) APT results when the interface of Mg rich side and steel was vertical, the all ion density map show a non-uniform density of ions, (b) rotation of the lifted out micro slab using the omni probe manipulator in the FIB and aligned the interface at 45 degree to the field ionization axis, (c) smooth transition from Fe rich side to the Mg rich side.

**Supplementary References**

1. Wang, K., et al. “Investigation of Interfacial Layer for Friction Stir Scribe Welded Aluminum to Steel Joints.” *J. Manuf. Sci. E.* **140**(11), 111005, <https://doi.org/10.1115/1.4040873> (2018).
2. Wang, T., Sidhar, H., Mishra, R. S., Hovanski, Y., Upadhyay, P., Carlson, B. “Evaluation of intermetallic compound layer at aluminum/steel interface joined by friction stir scribe technology.” *Mater. Des.* **174,** 107795, <https://doi.org/10.1016/j.matdes.2019.107795> (2020).
3. Wang, T., et al. “Effect of interfacial characteristics on magnesium to steel joint obtained using FAST,” *Mater. Des.* **192,** 108697, <https://doi.org/10.1016/j.matdes.2020.108697> (2020).
